# An independent validation of a clinical prediction rule for the diagnosis of cervical radiculopathy with radicular pain

**DOI:** 10.1016/j.bjpt.2026.101581

**Published:** 2026-05-02

**Authors:** Francis Grondin, Chad Cook, Toby Hall, Olivier Maillard, Yannick Perdrix, Sebastien Freppel

**Affiliations:** a Department of Neurosurgery, Saint-Pierre University Hospital, 97448 Saint Pierre, France; b Laboratory IRISSE, EA4075, Faculty of Human and Environment Sciences, University of La Réunion, Le Tampon, Réunion, France; c Physio Formation, Research and Development Department, Saint-Pierre, France; d Department of Orthopaedics, Duke University School of Medicine, Duke University, Durham, NC, USA; e Department of Population Health Sciences, Duke University Durham NC USA. Duke Clinical Research Institute, Duke University, Durham NC, USA; f Curtin School of Allied Health, Curtin University, Kent Street, Bentley, Perth, Australia; g Department of Public Health and Research, Clinical Investigation Center (CIC), INSERM CIC 1410, University Hospital, Reunion Island, 97410, Saint-Pierre, France; h University Hospital, Reunion Island, 97410, Saint-Pierre, France

**Keywords:** Accuracy, Arm pain, Neurodynamic tests, Posttest probabilities, Spurling test

## Abstract

**Background:**

A clinical prediction rule (CPR) for cervical radiculopathy (CR), published in 2003, is widely used in practice and recommended in clinical practice guidelines. To date, this CPR has not been independently validated.

**Objective:**

To perform an independent broad validation of the 2003 CPR using magnetic resonance imaging (MRI) as reference standard, and to investigate whether an alternative test cluster has stronger diagnostic utility for identifying CR with radicular pain

**Methods:**

This prospective diagnostic accuracy study was conducted following the updated STARD 2015 guidelines. Eighty-five individuals (27 with CR) were included from 109 consecutive patients. The diagnosis of CR was based on cervical spine MRI findings consistent with the patient’s clinical features, symptoms, and neurological examination, interpreted by a neurosurgeon. Twelve clinical tests were performed by the same examiner blinded to diagnosis.

**Results:**

Validation of the 2003 CPR (cervical distraction, Spurling’s test, upper limb neurodynamic test (ULNT) 1, and cervical rotation <60°) produced diagnostic values comparable to the original study. Our independent cluster based on backwards stepwise regression identified three variables: (1) modified (passive) shoulder abduction test, (2) Spurling’s arm pain test, and (3) ≥2 of 4 ULNTs positive. With this new cluster, having all three tests positive provided an infinite LR+ and a 100 % post-test probability, identifying 37.0 % of CR with radicular pain cases compared to 17.9 % in the original 2003 cluster (4/4 positive tests).

**Conclusion:**

This study findings corroborate the original 2003 CPR and identified a new cluster that had stronger diagnostic utility for CR with radicular pain.

## Highlights

• New 3-test cluster showed stronger diagnostic utility than original CPR.
• New cluster includes Spurling, Shoulder Abduction test, ≥2 positive ULNTs.
• Positive cluster (3/3) rules in CR with 100 % post-test probability.
• Negative (0/3) cluster rules out cervical radiculopathy diagnosis.
• Modified Shoulder Abduction test showed higher post-test probability.

## Introduction

Cervical radiculopathy (CR) comprises mechanical compression, neuropraxia, or chemical irritation of the nerve roots,^1^ resulting in characteristic signs and symptoms of arm pain, paresthesia, arm muscle weakness, reduced deep tendon reflexes, and exacerbation of symptoms with neck movements.^2^ Radicular patterns of symptoms vary depending on the involved nerve root, although some distributional overlap may exist.^3^ The condition is more common in individuals aged 20 to 50 years and is considered a ‘clinical diagnosis with imaging confirmation’, which is most frequently performed with magnetic resonance imaging (MRI).^4^^,^^5^

The North American Spine Society clinical practice guidelines,^6^ recommend a careful evaluation of patient history and clinical signs and symptoms, as well as the judicious use of clinical tests such as shoulder abduction sign (e.g., Bakody’s test) and Spurling’s test. Past reviews have advocated similar tests.^7^^,^^8^ However, considering the sensitivity and specificity of individual tests, authors reported limitations in using single test findings for diagnosis,^9^ a phenomenon known as “stand alone” weakness. Moreover, studies have reported that historical questions have poor diagnostic validity,^10^ justifying the use of validated combinations of physical tests to confirm the CR diagnosis. A past study built a clinical prediction rule (CPR),^10^ which combined test findings into a “single diagnostic test item cluster” to provide a more robust assessment of CR. The CPR included four tests: 1) upper limb neurodynamic test, 2) Spurling’s test, 3) cervical distraction, and 4) cervical rotation range of motion deficit on the side of radicular symptoms less than 60 degrees. To date, the CPR, which Wainner and colleagues published in 2003, has yet to be replicated and externally validated.

There are three main stages in the development of CPRs: 1) derivation, 2) external validation, and

3) impact analysis to determine their influence on patient care.^11^ Within the external validation stage, both narrow and broad validations are advocated.^11^ Narrow validation involves replication in a similar clinical setting, using a similar population of patients. Broad validation comprises testing in widely variable clinical settings, in populations with varying degrees of disease severity and prevalence. A majority of derived CPRs never reach the validation phase,^12^ or fail validation when tested especially in unique patient populations.^13^ The objectives of our study were 1) to perform an independent broad validation of the 2003 CPR,^10^ using clinical examination and MRI as the reference standard and 2) to investigate whether a novel cluster of tests had a stronger diagnostic utility. We hypothesized that despite a difference in reference standards (the 2003 paper used electromyography as a reference standard), the CPR will still demonstrate strong diagnostic accuracy when discriminating CR and would represent the strongest cluster of tests evaluated.

## Methods

### Study design

The study was a prospective diagnostic accuracy study with a consecutive cohort of patients presenting with neck pain and/or radicular symptoms (radicular pain, paresthesia, sensory or motor deficits)**.** All diagnostic decisions were made under conditions of diagnostic uncertainty. The non-CR group in our design represents individuals in which the clinician would differentially diagnose in a true given clinical setting. To improve the transparency of reporting, we followed the updated 2015 Standards for Reporting Diagnostic accuracy studies (STARD).^14^ The study was conducted in accordance with the ethical principles and the Helsinki Declaration on research involving human subjects and was approved (n°2212189v0).

### Participants

**Fig. 1:**
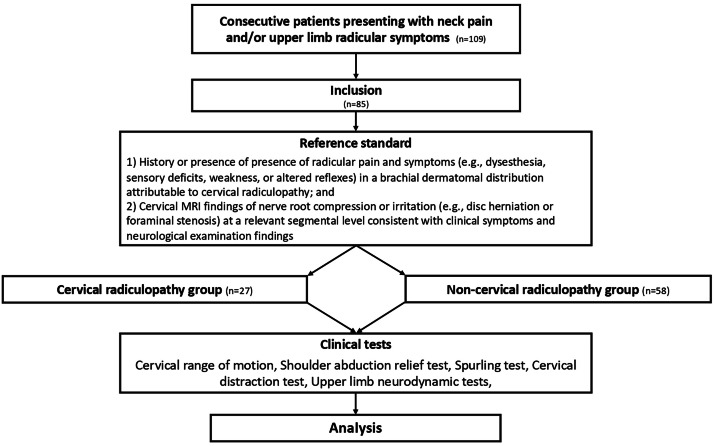
Consort flow of study patients.

Participants included consecutive patients presenting with neck pain and/or radicular symptoms, referred by a general practitioner or specialist between September 2017 and September 2019 to a Neurosurgery Department. This unfiltered recruitment approach was intended to reflect the clinical heterogeneity typically encountered in routine practice. All participants received the same diagnostic work-up, including an MRI, and were included for analysis (Fig. 1). Inclusion required reporting neck pain and/or arm pain of at least 3-months in duration, age 18 to 65 years, self-report of pain of at least 30 and less than 80 on a 100 mm visual analogue scale (VAS)^17^^,^^18^ during the previous 24 hours, self-report of a score of ≥20 % on the Neck Disability Index questionnaire (NDI),^17^^,^^19^ and symptoms of at least 3-months in duration. Potential subjects were excluded if they had suffered significant neck trauma at the time of the study, had a history of neck or arm surgery, inflammatory joint condition or arthritis, fibromyalgia, diabetes, psychiatric disorders, pregnancy, cardiovascular or neoplastic pathology, cervical myelopathy, pyramidal or extrapyramidal pathology, or were unable to speak or write in French.

## Test methods

### Index tests

We analyzed 12 clinical tests or findings from the patient history in comparison to the diagnostic reference standard. No intervention occurred between the index test(s) and reference standard, which were performed on the same day. The clinical tests were the same as those previously studied by Wainner et al’s including all four upper limb neurodynamic tests (ULNT). The same physical therapist with 10 years of experience in neck pain management, and advanced certification for orthopedic assessment performed the clinical tests one hour after the clinical/MRI diagnosis. The physical therapist conducting the clinical tests was blinded to the patient's medical history and final diagnostic conclusions. Clinical tests included a modified shoulder abduction relief test (Bakody’s sign), performed passively by maintaining the patient’s hand on the head for 5 seconds to avoid pain from active execution (e.g., shoulder pain, apprehension). Spurling’s test with reproduction of familiar neck symptoms, Spurling’s test with reproduction of familiar arm symptoms. The cervical distraction test (∼14 kg for 10 s),^10^ and four upper limb neurodynamic tests (ULNT1, ULNT 2a, ULNT 2b, and ULNT3),^15^ with reproduction of familiar symptoms and structural differentiation,^20^^,^^21^ cervical rotation <60°, neck range of motion ipsilateral<contralateral, age >48 years, duration of symptoms<62 weeks, which are described in the online material.

The age and duration of symptoms cut offs were recommended after using area under the curve threshold assessments. Cut offs were determined with duration of symptoms <62 weeks, and age >48 years. Tests were considered positive or negative if they met the preset criteria (online material), and indeterminate if the patient was unable to tolerate the test position to allow test completion.

### Reference standard

The diagnosis of CR or a competing diagnosis was made by a single neurosurgeon, who was masked to the results of the 12 clinical test findings (which occurred after the diagnosis and were not part of the neurosurgeon’s examination). The surgeon had 15 years of experience and based the diagnosis of CR on the following criteria: 1) history and presence of dermatomal radicular pain and/or symptoms (dysesthesia, sensory deficit, muscle weakness, or altered reflexes) attributable to a CR and 2) presence of MRI findings with nerve root compression or irritation due to disc herniation or foraminal stenosis at a relevant segmental level (i.e., the same or adjacent level) on the ipsilateral side consistent with the patient’s symptoms and neurological examination findings.^4^

## Analysis

Statistical analyses were performed using IBM SPSS version 26.0 Statistics for Windows, version 26 (IBM Corp., Armonk, N.Y., USA). Descriptive statistics were calculated as means and standard deviations (SD), frequencies, and percentages were tabulated to describe the included participants. Differences among those diagnosed with CR and those with competing conditions were evaluated with Wilcoxon rank-sum tests for continuous variables and chi-square or Fisher exact for categorical variables. All 12 clinical tests and the condition of 1 of 4, 2 of 4, 3 of 4, and 4 of 4 positive ULNT were individually examined for diagnostic accuracy against the MRI reference standard. Contingency tables (2 × 2) were used to calculate sensitivity and specificity, likelihood ratios (positive likelihood ratio [LR+]; negative likelihood ratio [LR−]), and posttest probabilities with a positive and negative finding for each single test. Receiver operating characteristic (ROC) curves were used to determine all previously mentioned possible cut‐off values for age and symptoms duration. Sensitivity was defined as the proportion of individuals with cervical radiculopathy who had a positive test, while specificity was defined as the proportion of individuals without cervical radiculopathy who had a negative test. LR+ and LR− were calculated using the following formula:

*LR+ = sensitivity / (1 − specificity); LR− = (1 − sensitivity) / specificity.*

Post-test probability was calculated using Bayes’ theorem, based on the pre-test probability and LR:

*– Post-test probability with a positive finding = (pre-test probability × LR+) / [(pre-test × LR+) + (1 - pre-test)]*

*– Post-test probability with a negative finding = (pre-test probability × LR-) / [(pre-test × LR-) + (1 - pre-test)]*

We ran two independent cluster analyses to test our two objectives.

For our first objective (broad validation of the 2003 CPR), we included the same clinical tests (upper limb neurodynamic test, Spurling’s test, cervical distraction, and range of motion deficit to the side of symptoms less than 60 degrees) that were used by Wainner and colleagues.^10^ Using a similar strategy, we clustered the findings into conditions of 1 of 4, 2 of 4, 3 of 4, and 4 of 4 positive findings. Independent diagnostic accuracy values were run for each condition (e.g., 1 of 4, 2 of 4, *etc.*).

For our second objective (creation of a novel CPR), we identified the conditionally independent variables from the individual 2 × 2 analyses that yielded LR+ above 1.5 or LR− below 0.5 for a backward stepwise logistic regression analysis. This analysis was used to select variables, with p values of 0.15 to exit the model and 0.10 to enter it. This is a similar strategy to that used by Wainner and colleagues in 2003. Variables retained by the regression model were used to cluster findings and were then inputted into similar conditions (e.g., 1 of N, 2 of N, etc.). For each condition, sensitivity, specificity, positive and negative likelihood ratios, and post-test probabilities for both positive and negative findings were analyzed.

## Results

Our study included 85 patients, including 31.7 % (n = 27) diagnosed with CR, whereas the non-CR group included 58 people, 42 of whom presented with neck without CR, 12 with peripheral nerve entrapment, and 4 with diffuse shoulder pain. The CR group presented with a significantly higher proportion of females and a longer duration of symptoms versus the non-CR group (Table 1). No adverse events from performing the index test or the reference standard were reported. The individual diagnostic accuracy values of our 12 pre-selected clinical tests are summarized in Table 2. The highest sensitivity was observed with one of the four ULNT tests, which had a sensitivity of 96.3 % and reached a post-test probability with a positive finding of 45.1 %. In contrast, the Spurling test demonstrated the highest specificity, with a positive likelihood ratio (LR+) of 34.37 (95 %CI = 4.80, 245.98) and a post-test probability with a positive finding of approximately 94.10 %. The strongest individual test for ruling out was cervical distraction, in which a negative test resulted in a post-test probability of 11.86 %. Table 3 explores the broad validation of Wainner’s 2003 cluster,^10^ with the four following tests: 1) cervical distraction, 2) Spurling’s, 3) ULNT 1, and 4) cervical range of motion less than 60° to the ipsilateral side. Our findings show that the condition of 1 out of 4 positive tests was the most sensitive combination whereas the condition of 4 out of 4 four tests was the most specific. When none of the tests were positive, it ruled out all cases of CR (LR-=0.00; post-test probability = 0 %). The condition of having four out of four positive test results yielded an infinite LR+ and a post-test probability of 100% for confirming the diagnosis of cervical radiculopathy, however, having all four positive tests identified 17.9 % of patients with CR (Table 3).

**Table 1:** Sampling statistics.

	Cervical radiculopathies (CR) (n=27)	Non CR-group (n=58)
Age	43.96 (8.94)	45.27 (9.74)
Sex	12 males/15 females	13 males/45 females
Side (right)	12 right ; 15 left	33 right ; 25 left
Body Mass Index (kg/m²)	24.73 (3.91)	24.88 (4.97)
Duration of symptoms (self-report) in months	93.25 (98.41)	70.51 (62.31)
Neck Disability Index (%)	38.16 (14.14)	43.07 (13.90)
Visual Analog Scale for Pain (0–100 mm)	51.4 (15.8)	50.3 (15.3)

* Statistically significant difference between CR and non-CR groups (*p* < 0.05)

CR: Cervical radiculopathy

**Table 2:** Sensitivity, specificity, likelihood ratios, and post-test probabilities of single test findings (Pre-test prevalence = 31.7 %).

**Test**	**Sensitivity (95% CI)**	**Specificity (95% CI)**	**LR+ (95% CI)**	**LR- (95% CI)**	**Posttest probability with a positive finding**	**Posttest probability with a negative finding**
**Shlouder Abduction test**	77.78 (57.74, 91.38)	62.07 (48.37, 74.49)	2.05 (1.39, 3.02)	0.36 (0.17, 0.75)	48.85 (39.21, 58.36)	14.31 (7.31, 25.82)
**Cervical distraction**	88.89 (70.84, 97.65)	37.93 (25.51, 51.63)	1.43 (1.12,1.82)	0.29 (0.10, 0.89)	39.89 (34.20 45.79)	11.86 (4.43, 29.23)
**Spurling’s (Neck)**	70.37 (49.82, 86.25)	58.62 (44.93, 71.40)	1.70 (1.15, 2.52)	0.51 (0.27, 0.94)	44.10 (34.80, 53.90)	19.14 (11.13, 30.37)
**Spurling’s (Arm)**	59.26 (38.80, 77.61)	98.28 (90.76, 9.96)	34.37 (4.80, 245.98)	0.41 (0.26, 0.65)	94.10 (69.01, 99.13)	15.98 (10.76, 23.17)
**ULNT 1**	59.26 (38.80, 77.61)	75.86 (62.83, 86.13)	2.46 (1.41, 4.27)	0.54 (0.33, 0.87)	53.30 (39.55, 66.46)	20.04 (13.28, 28.76)
**ULNT 2a**	70.37 (49.82, 86.25)	72.41 (59.10, 83.34)	2.55 (1.5, 4.14)	0.41 (0.22, 0.75)	54.20 (42.15, 65.77)	15.98 (9.26, 25.82)
**ULNT 2b**	55.56 (35.33, 74.52)	75.86 (62.83, 86.13)	2.30 (1.30, 4.06)	0.59 (0.38, 0.92)	51.63 (37.63, 65.33)	21.49 (14.99, 29.92)
**ULNT 3**	40.74 (22.39, 61.20)	93.10 (83.27, 98.09)	5.91 (2.07, 16.87)	0.64 (0.46, 0.88)	73.28 (48.99, 88.65)	22.95 (17.59, 28.99)
**Cervical rotation <60°**	62.96 (42.37, 80.60)	50.00 (36.58, 63.42)	1.26 (0.85, 1.85)	0.74 (0.43, 1.29)	36.90 (28.29, 46.19)	25.56 (16.63, 37.45)
**Cervical rotation ROM Ipsilateral < Contralateral**	33.33 (16.52, 53.96)	74.14 (60.96, 84.74)	1.29 (0.65, 2.57)	0.90 (0.66, 1.22)	37.45 (23.17, 54.39	29.46 (23.44, 36.15)
**Age >48 years**	29.63 (13.75, 50.18)	60.34 (46.64, 72.95)	0.75 (0.39, 1.45)	1.17 (0.85, 1.61)	25.82 (15.32, 50.22)	35.19 (28.29, 42.76)
**Duration of symptoms <62 weeks**	55.56 (35.33, 74.52)	46.55 (33.34, 60.13)	1.04 (0.69, 1.57)	0.95 (0.58, 1.58)	32.55 (24.25, 42.15	30.60 (21.20, 42.30)
**1 of 4 ULNT positive**	96.30 (81.03, 99.91)	46.55 (33.34, 60.13)	1.80 (1.40, 2.32)	0.08 (0.01, 0.56)	45.51 (39.38, 51.84)	3.58 (0.46, 20.62)
**2 of 4 ULNT** positive	85.19 (66.27, 95.81)	74.14 (60.96, 84.74)	3.29 (2.07, 5.23)	0.20 (0.08, 0.50)	60.42 (48.99, 70.82)	8.49 (3.59, 18.83)
**3 of 4 ULNT positive**	44.44 (25.48, 64.67)	96.55 (88.09, 99.58)	12.89 (3.10, 53.62)	0.58 (0.41, 0.81)	85.71 (59.06, 96.14)	23.24 (18.57, 28.82)

SPECIFICITY (95 % CI)

LR+

(95 % CI)

LR-

(95 % CI)

Posttest probability with a positive finding

Posttest probability with a negative finding

Pre-test prevalence = 31.7 %

95 % CI: 95 % confidence interval

Inf: Infinite

ULNT: Upper Limb Neurodynamic test

ROM: Range of motion

**Table 3:** Diagnostic accuracy of Wainner et al.’s CPR – performance of the original test cluster.

Conditions	Sensitivity (95 % CI)	Specificity (95 % CI)	LR+ (95 % CI)	LR- (95 % CI)	Post-test probability with a positive finding (95% CI)	Post-test probability with a negative finding (95% CI)
One of four tests positive	100 (88.8, 100)	15.8 (10.3, 15.8)	1.18 (0.98, 1.19)	0.00 (0.00, 1.09)	35.39% (31.26, 35.58)	0.00% (0.00, 33,59)
Two of four tests positive	82.1 (65.7, 92.9)	59.6 (57.6, 64.9)	2.04 (1.36, 2.64)	0.29 (0.11, 0.66)	48.63% (38.7, 55,06)	11.86% (4.86, 23.45)
Three of four tests positive	67.9 (52.7, 78.1)	91.2 (83.8, 96.2)	7.73 (3.24,20.74)	0.35 (0.23, 0.56)	78.20% (60.06, 90.59)	13.97% (9.65, 20.63)
Four of four tests positive	17.9 (8.5, 17.9)	100(95.4, 100)	Inf (1.85, Inf)	0.82 (0.82, 0.96)	100.00% (46.20 , 100.0)	27.57% (27.57, 30.82)

Four tests include (1) cervical distraction, (2) Spurling’s, (3) Upper Limb Neurodynamic test 1, (4) Cervical rotation range of motion less than 60° to ipsilateral side.

Pre-test prevalence = 31.7 %

95 % CI: 95 % confidence interval

Inf: Infinite

LR+: Positive likelihood ratio

LR-: Negative likelihood ratio

For our second objective, we evaluated an independent cluster using the test findings. Eight of the tests (shoulder abduction, cervical distraction, Spurling’s neck pain, Spurling’s arm pain, and the four ULNT) met our a priori criteria for inclusion in the backward stepwise regression.

This analysis identified three variables that were included in the final model: 1) Modified shoulder abduction test, 2) Spurling’s arm pain test, and 3) 2 of 4 positive ULNT. The condition '2 out of 4 ULNTs’ was considered as a single composite variable, meaning any two positive results among the four described ULNTs. When none of the tests were positive, it ruled out all cases of CR (posttest probability=0 %). Having three out of three tests positive led to an infinite LR+ and a post-test probability of 100 % for confirming the diagnosis of cervical radiculopathy. When all three of the conditions were positive, no individuals without radiculopathy were identified as positive (post-test probability=100 %). Two of three positive tests yielded a LR+ of 7.06 (95 %CI=3.63, 12.20) and a LR- of 0.17 (95 %CI=0.06, 0.37) (Table 4).

**Table 4:** Diagnostic accuracy of the novel clinical test cluster.

Conditions	Sensitivity(95 % CI)	Specificity (95 % CI)	LR+(95 % CI)	LR-(95 % CI)	Posttest probability with a positive finding	Posttest probability with a negative finding
One of three tests positive	100 (86.6, 100)	43.10 (36.8, 43.1)	1.75 (1.37, 1.75)	0.00 (0.00, 0.35)	44.82 % (38.87, 44.82)	0.00 % (0.00, 13.97)
Two of three tests positive	85.2 (69.8, 94.5)	87.9 (80.8, 92.3)	7.06 (3.63, 12.20)	0.17 (0.06, 0.37)	76.62 % (62.75, 84.99)	7.31 % (2.71, 14.66)
Three of three tests positive	37.0 (25.2, 37.0)	100 (94.5, 100.0)	Inf (4.57, Inf)	0.63 (0.63, 0.79)	100.00 % (67.96, 100)	22.62 % (22.62, 26.83)

Pre-test prevalence = 31.7 %

Three tests include (1) shoulder abduction, (2) Spurling’s arm, and (3) at least 2 ULNT positive.

The condition 'at least 2 Upper Limb neurodynamic tests positive' was considered as a single composite variable, meaning any two positive results among the four described ULNTs

95 % CI: 95 % confidence interval

Inf: Infinite

LR+: Positive likelihood ratio

LR−: Negative likelihood ratio

## Discussion

Since the 2003 publication,^10^ Wainner et al.’s clinical prediction rule for CR has been included in clinical practice guidelines,^16^ and is used frequently in clinical practice settings. The study has been cited over 500 times but, to date, has not been independently (externally) validated in a separate study by a different authorship group. Our study objectives were to 1) broadly validate the CR clinical prediction rule and 2) evaluate whether another rule was as effective or more effective than the 2003 rule created by Wainner and colleagues.^10^ We were able to accomplish both tasks.

The broad validation of the 2003 CPR (Distraction, Spurling’s test, Ipsilateral cervical rotation <60°, and ULNT1),^10^ resulted in very similar diagnostic metrics for all categories. In fact, our results yielded a slightly better posttest probability with 2, 3, or 4 positive conditions (48.6 %, 78.2 %, and 100 %, respectively) compared to those reported by Wainner et al.^10^ (21 %, 65 %, and 90 %, respectively) (Table 4).

Based on Wainner's test cluster, the probability increases to approximately 49 %, which is insufficient to establish a diagnosis. However, a diagnosis of cervical radiculopathy can be considered when at least three out of four tests are positive (78.2 %). Furthermore, when none of the four test findings were positive in our study, it completely ruled out the presence of CR (post-test probability=0 %). Wainner and colleagues did not report the LR- of their cluster nor did they calculate a post-test probability when none of the findings were present.^10^ Our results suggest that the CPR demonstrated both rule-in and rule-out utility when dedicated conditions (i.e., 1 of 4 versus 4 or 4) are evaluated. Whereas minor variations in diagnostic metrics are expected when examining a completely different study sample, we feel that other factors that are unique to our methodology are also worth reporting. For example, a positive test finding in our study for Spurling’s arm pain/symptoms test included reproduction of familiar arm signs or symptoms. Our results with a LR+ of 34.37 are consistent with previous studies reporting values between 6.75,^22^ 8.93,^23^ to infinity,^24^ particularly when peripheral radicular pain or symptoms were provoked. While Wainner and colleagues defined Spurling’s A as positive when symptoms were reproduced (either in the arm or the neck),^10^ we analyzed these two symptoms reproduction separately. Our results suggest that arm symptom reproduction has greater diagnostic value for cervical radiculopathy with radicular pain. Reproduction of familiar neck pain alone resulted in a poor LR+ (1.70) and posttest probability with a positive finding (44.1 %), which is also similar to past findings based on non-specific “symptoms reproduction” of the Spurling test, with LR+ ranging from 0.93,^25^ 2.1,^10^ to 6.75.^22^ Our definition of a positive ULNT test and the combined ULNT tests are also unique to our study. For those diagnosed with CR, we found that 85 % had 2 of 4 ULNT positive findings whereas 37 % of patients with CR had 3 of 4 ULNT positive findings. Previous evidence suggested that a fully negative ULNT profile is rare in patients with cervical radiculopathy and radicular symptoms, and no individual ULNT has demonstrated clear diagnostic superiority for CR diagnosis.^21^ In our study, the variable “≥2 ULNTs positive” — retained in the regression model — exhibited greater diagnostic utility than single ULNTs, particularly when interpreted alongside other clinical tests. Based on this finding, we would recommend the use of a ULNT cluster when assessing the potential for CR. To the best of our knowledge no previous published study assessed this condition of 2 of 4 ULNT positive findings in a cluster of tests for CR diagnosis.

Our second objective was to explore whether a separate combination of findings led to a better diagnostic cluster than Wainner’s original tool.^10^ While our findings corroborate the original 2003 CPR, we also identified a new cluster that yielded slightly better post-test probability results than the replication of Wainner et al.'s CPR. Our best combination of tests incorporated: 1) the Modified shoulder abduction test, 2) Spurling’s arm pain test, and 3) at least two of four ULNT positive. As with the Wainner CPR, having all four tests negative rules out a CR diagnosis (post-test probability of 0 %). When all the tests were positive one is also able to rule in CR, with a post-test probability of 100 %.

One potential benefit of our cluster, despite having to perform up to six tests (shoulder abduction, Spurlings’s, ULNT1, ULNT2a, ULNT2b, and ULNT3) versus four tests in Wainner’s cluster (range of motion, Spurlings’s, Distraction and ULNT1) is the number of individuals with CR that are correctly identified with our combination. Over 37 % of individuals with CR were captured when 3 of 3 conditions were positive in our study (100 % post-test probability), whereas only 17.9 % of with Wainner’s 4 of 4 cluster (100 % post-test probability). This suggests that many subjects are missed in Wainner’s rule that are less likely misdiagnosed in our cluster.

Although the Shoulder Abduction relief test (Bakody’s sign) has been commonly described in the past in CR diagnosis,^26^, ^27^, ^28^ few studies have assessed its diagnostic validity. Previous studies reported similar LR+ (Wainner 2.1; Sleijser-Koehorst 1.88; Viikari-Juntura 3.03; Gashemi 1.33). Previous studies reported sensitivities that have ranged from 17 % (Wainner) to 50 % (Sleijser-Koehorst) in comparison with 77.78 % in our study. The shoulder abduction test was performed differently in our study versus previous studies by Gashemi et al,^25^ Viikari-Juntura et al,^22^ Sleijser-Koehorst et al,^28^ and Wainner et al,^10^ as we had the examiner passively maintain the hand on the patient’s head for 5 seconds. This may be the reason for the increased sensitivity findings in our study, which influenced the combined modeling of multiple tests.

Consideration needs to be given to the relative merit of clinical tests for relief and provocation of neck and arm symptoms. Some patients with CR may not have arm pain or symptoms at rest, hence achieving a reduction in symptoms is not possible during selected relief tests such as the neck distraction and shoulder abduction test. This might explain why relief of neck or arm symptoms are included as indicative of a positive test for both the Shoulder abduction test and Spurling’s test.^10^ This may also explain why tests evaluating symptom relief are more sensitive and provocation tests (Spurling‘s test and ULNT) were more specific. Further studies are necessary to investigate the accuracy of different criteria for positive tests in patients with CR with varying severity of neck and/or arm symptoms.

The disparity in pre-test probability between our study (32 %) and Wainner's study (23 %) may be explained by differences in inclusion criteria (not detailed) and the use of EMG as the gold standard (versus clinical examination and MRI), which can yield negative results if performed before denervation or after complete reinnervation.^29^ We aimed to minimize pre-selection bias and reported a pre-test prevalence of CR (31.7 %) similar to that observed by Wainner et al. (23 %) in primary care settings.^10^ Although our novel cluster demonstrated clinically meaningful diagnostic performance — with a LR− < 0.1 (strong rule-out) when all tests were negative and an infinite LR+ (definitive rule-in) when all were Positive —^30^ post-test probabilities should always be interpreted within the clinical context. These values may differ in settings with higher pre-test prevalence, such as multidisciplinary referrals (49 %),^28^ or electrodiagnostic testing (79 %).^25^

A potential limitation of our study is the small sample size of 85 (including 27 people with CR), which may lead to less precision (e.g., wide confidence intervals). More impaired cases on the spectrum of CR might be represented in our sample.^31^^,^^32^ The reference standard was determined by only one clinician while another evaluated all clinical tests. Although the clinical tester was blinded to the diagnosis of the patient, the transferability of their findings is unknown, since we did not test interrater agreement related to the index tests studied. This new cluster should also be interpreted with caution due to the regression model employed. Backwards stepwise regression method has the potential for unstable selection and overfitting.^33^ Further validation of this new cluster is required with a larger sample of patients with CR in diverse clinical settings (with no radicular pain, with posterolateral or foraminal or lateral recess radiculopathy), and larger non-CR group populations (cervicobrachial pain, thoracic outlet syndrome, *etc.*), and with more examiner and reference standards including those evaluating nerve small fiber function and other forms of imaging such as magnetic resonance neurography.

## Conclusion

Our findings broadly and independently validate the 2003 CPR for CR by Wainner and colleagues,^10^ and support its continued use in clinical practice. We were also able to identify a unique diagnostic cluster for CR with radicular pain that involved 1) a modified shoulder abduction test for arm pain relief, 2) Spurling’s test for arm symptoms provocation, and 3) at least 2 positive ULNTs. This new cluster ruled out all cases of CR when none of the tests were positive (post-test probability = 0 %) and it ruled in the CR diagnosis when two of three tests were positive (LR+ of 7.13 and post-test probability with a positive finding of 76.79 %). When all three criteria are positive the LR+ is infinite and post-test probability with a positive finding 100 %.

## Declaration of competing interest

The authors declare no competing interest.

## Appendix Supplementary materials

Image, application 1

## References

[1] Woods B.I., Hilibrand A.S. (2015). Cervical radiculopathy: epidemiology, etiology, diagnosis, and treatment. J Spinal Disord Tech.

[2] Iyer S., Kim H.J. (2016). Cervical radiculopathy. Curr Rev Musculoskelet Med.

[3] Eubanks J.D. (2010). Cervical radiculopathy: nonoperative management of neck pain and radicular symptoms. Am Fam Physician.

[4] Carette S., Fehlings M.G. (2005). Clinical practice: cervical radiculopathy. N Engl J Med.

[5] Kuijper B., Tans J.T., Schimsheimer R.J. (2009). Degenerative cervical radiculopathy: diagnosis and conservative treatment. A review. Eur J Neurol.

[6] Bono C.M., Ghiselli G., Gilbert T.J. (2011). An evidence-based clinical guideline for the diagnosis and treatment of cervical radiculopathy from degenerative disorders. Spine J.

[7] Thoomes E.J., van Geest S., van der Windt D.A. (2018). Value of physical tests in diagnosing cervical radiculopathy: a systematic review. Spine J.

[8] Rubinstein S.M., Pool J.J., van Tulder M.W., Riphagen I.I., de Vet H.C. (2007). A systematic review of the diagnostic accuracy of provocative tests of the neck for diagnosing cervical radiculopathy. Eur Spine J.

[9] Cowley L.E., Farewell D.M., Maguire S. (2019). Methodological standards for the development and evaluation of clinical prediction rules: a review of the literature. Diagn Progn Res.

[10] Wainner R.S., Fritz J.M., Irrgang J.J., Boninger M.L., Delitto A., Allison S. (2003). Reliability and diagnostic accuracy of the clinical examination and patient self-report measures for cervical radiculopathy. Spine (Phila Pa 1976).

[11] McGinn T. (2016). Putting meaning into meaningful use: a roadmap to successful integration o evidence at the point of care. JMIR Med Inform.

[12] Cook C.E. (2008). Potential pitfalls of clinical prediction rules. J Man Manip Ther.

[13] Haskins R., Cook C. (2016). Enthusiasm for prescriptive clinical prediction rules (eg, back pain and more): a quick word of caution. Br J Sports Med.

[14] Bossuyt P.M, Reitsma J.B, Bruns D.E (2015). STARD Group. STARD 2015: an updated list of essential items for reporting diagnostic accuracy studies. Radiology.

[15] Nee R.J., Jull G.A., Vicenzino B., Coppieters M.W. (2012). The validity of upper-limb neurodynamic tests for detecting peripheral neuropathic pain. J Orthop Sports Phys Ther.

[16] Childs J.D., Cleland J.A., Elliott J.M. (2008). Neck pain: clinical practice guidelines linked to the international classification of functioning, disability, and health from the orthopaedic section of the American physical therapy association. J Orthop Sports Phys Ther.

[17] Young I.A., Dunning J., Butts R., Mourad F., Cleland J.A. (2019). Reliability, construct validity, and responsiveness of the neck disability index and numeric pain rating scale in patients with mechanical neck pain without upper extremity symptoms. Physiother Theory Pract.

[18] Guo Q., Zhang L., Han L.L. (2024). Effects of virtual reality therapy combined with conventional rehabilitation on pain, kinematic function, and disability in patients with chronic neck pain: Randomized controlled trial. JMIR Serious Games.

[19] Cetin H., Kose N., Oge H.K. (2022). Virtual reality and motor control exercises to treat chronic neck pain: a randomized controlled trial. Musculoskelet Sci Pract.

[20] Verhagen A.P., Brown H., Hancock M., Anderson D. (2023). Test procedures and positive diagnostic criteria of the upper limb tension tests differ: a systematic review of the DiTA database. Braz J Phys Ther.

[21] Grondin F., Cook C., Hall T., Maillard O., Perdrix Y., Freppel S. (2021). Diagnostic accuracy of upper limb neurodynamic tests in the diagnosis of cervical radiculopathy. Musculoskelet Sci Pract.

[22] Viikari-Juntura E., Porras M., Laasonen E.M. (1989). Validity of clinical tests in the diagnosis of root compression in cervical disc disease. Spine (Phila Pa 1976).

[23] Shabat S., Leitner Y., David R., Folman Y. (2012). The correlation between Spurling test and imaging studies in detecting cervical radiculopathy. J Neuroimaging.

[24] Shah K.C, Rajshekhar V. (2004). Reliability of diagnosis of soft cervical disc prolapse using Spurling's test. Br J Neurosurg.

[25] Ghasemi M., Golabchi K., Mousavi S.A., Asadi B., Rezvani M., Shaygannejad V., Salari M. (2013). The value of provocative tests in diagnosis of cervical radiculopathy. J Res Med Sci.

[26] Davidson R.I., Dunn E.J., Metzmaker J.N. (1981). The shoulder abduction test in the diagnosis of radicular pain in cervical extradural compressive monoradiculopathies. Spine (Phila Pa 1976).

[27] Fast A., Parikh S., Marin E.L. (1989). The shoulder abduction relief sign in cervical radiculopathy. Arch Phys Med Rehabil.

[28] Sleijser-Koehorst M.L.S., Coppieters M.W., Epping R., Rooker S., Verhagen A.P., Scholten-Peeters G.G.M. (2021). Diagnostic accuracy of patient interview items and clinical tests for cervical radiculopathy. Physiotherapy.

[29] Ashkan K., Johnston P., Moore A.J. (2002). A comparison of magnetic resonance imaging and neurophysiological studies in the assessment of cervical radiculopathy. Br J Neurosurg.

[30] Jaeschke R., Guyatt G.H., Sackett D.L. (1994). Evidence-based medicine working group. Users' guides to the medical literature. III. How to use an article about a diagnostic test. B. What are the results and will they help me in caring for my patients? The evidence-based medicine working group. JAMA.

[31] Rutjes A.W., Reitsma J.B., Di Nisio M., Smidt N., van Rijn J.C., Bossuyt P.M. (2006). Evidence of bias and variation in diagnostic accuracy studies. CMAJ.

[32] Hall M.K., Kea B., Wang R. (2019). Recognising bias in studies of diagnostic tests. Part 1: patient selection. Emerg Med J.

[33] Grant S.W., Collins G.S., Nashef S.A.M. (2018). Statistical primer: developing and validating a risk prediction model. Eur J Cardiothorac Surg.

