# Supplementary material for: An independent validation of a clinical prediction rule for the diagnosis of cervical radiculopathy with radicular pain
Source: Braz J Phys Ther. 2026 May 2;30(3):101581. doi: 10.1016/j.bjpt.2026.101581 (PMC13147772; doi:10.1016/j.bjpt.2026.101581)
Supplement: Supplementary file 1 [file mmc1.docx]

**Online material**: Test Descriptions.

| Test | Test Description |  |
| --- | --- | --- |
| **CERVICAL ROTATION <60°** |  | **Procedure**  While seated, the patient actively and maximally rotated their head until firm resistance or symptoms/pain occurred. Examiner measured the maximal rotation range of motion with a compass App built into an Iphone. The phone was fixed in a horizontal position (as indicated by the compass) to the apex of the participant's head with Velcro straps. |
|  |  | **Criteria for positive test**  Cervical rotation less than 60° toward the side of neck or arm symptoms |
| **SPURLING TEST  NECK SYMPTOMS** | **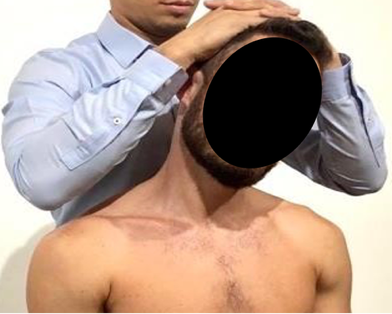** | **Procedure**  While seated, the patient is guided into side flexion to a maximum of 30°, or less if neck or arm symptoms occur on the ipsilateral side. The examiner then applies a progressive vertical compression force (~7 kg) to the top of the patient’s head for 5 seconds. |
|  |  | **Criteria for positive test:**  Provocation of familiar neck pain/symptoms |
| **SPURLING TEST  ARM SYMPTOMS** | **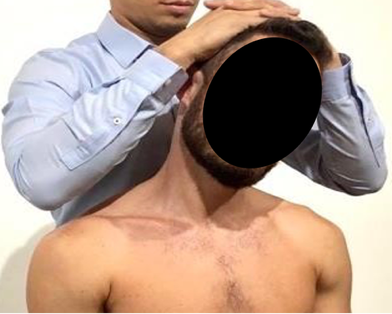** | **Procedure**  (Same procedure as above) |
|  |  | **Criteria for positive test**  Provocation of familiar arm pain or symptoms |
| **BAKODY’S SIGN  (SHOULDER ABDUCTION TEST)** | **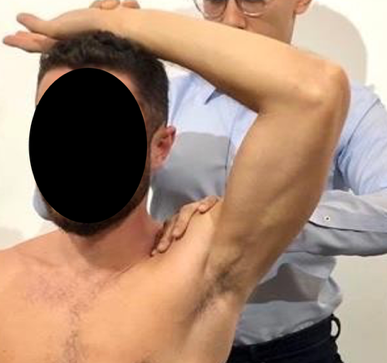** | **Procedure**  While seated, the patient’s hand on the affected side was passively placed on top of the head by the examiner. The patient was instructed to relax the arm and shoulder. The examiner passively maintained the hand without applying pressure on the patient’s head for 5 seconds |
|  |  | **Criteria for positive test**  Reduction in familiar shoulder/arm pain and/or neurological symptoms. |
| **NECK DISTRACTION** | 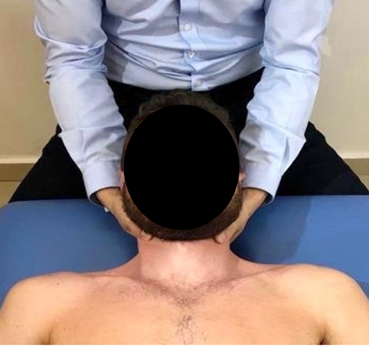 | **Procedure**  A traction force of approximately 14 kg was applied to the head via the occiput and maintained for 5 seconds by the examiner. |
|  |  | **Criteria for positive test**  Reduction in familiar neck, shoulder, arm pain and/or neurological symptoms. |
| **ULNT 1 (MEDIAN NERVE)** | 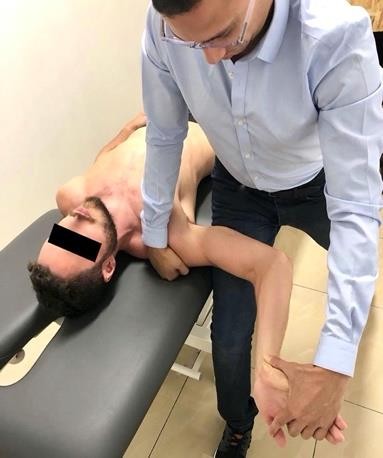 | **Procedure**  Shoulder girdle stabilization, shoulder abduction,  wrist/finger extension, forearm supination, shoulder  external rotation, elbow extension, |
|  |  | **Criteria for positive test**  Provocation of familiar neck, shoulder, or arm symptoms (neurological or pain) during the procedure, combined with structural differentiation through cervical side bending, release of shoulder girdle depression, or release of wrist extension, depending on the location of the provoked symptoms |
| **ULNT 2a (MEDIAN NERVE)** | 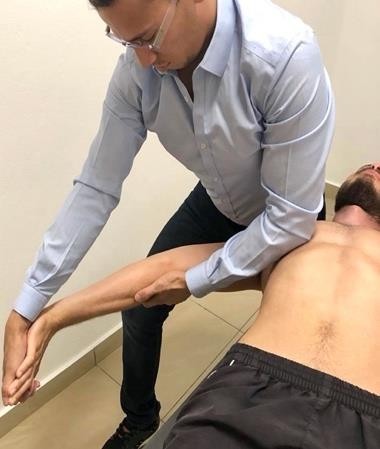 | **Procedure**  Shoulder girdle depression, elbow extension, shoulder external rotation and forearm supination, wrist/finger extension, shoulder abduction |
|  |  | **Criteria for positive test**  Provocation of familiar neck, shoulder, or arm symptoms (neurological or pain) during the procedure, combined with structural differentiation through cervical side bending, release of shoulder girdle depression, or release of wrist extension, depending on the location of the provoked symptoms. |
| **ULNT 2b  (RADIAL NERVE)** | 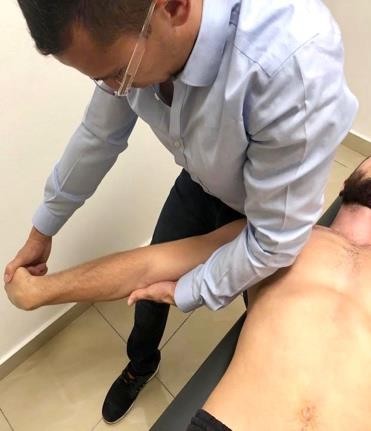 | **Procedure**  Shoulder girdle depression, elbow extension, shoulder external rotation, forearm pronation, wrist/finger flexion, shoulder abduction |
|  |  | **Criteria for positive test**  Provocation of familiar neck, shoulder, or arm symptoms (neurological or pain) during the procedure, combined with structural differentiation through cervical side bending, release of shoulder girdle depression, or release of wrist extension, depending on the location of the provoked symptoms. |
| **ULNT 3 (ULNAR NERVE)** | 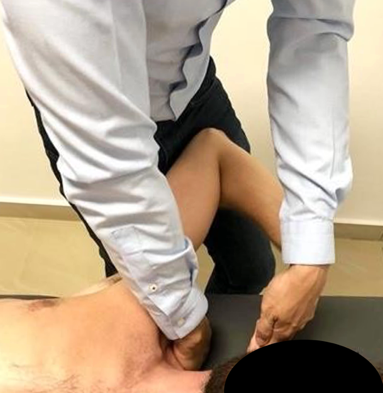 | **Procedure**  Wrist/finger extension, forearm pronation, elbow flexion, shoulder external rotation, shoulder girdle depression, shoulder abduction |
|  |  | **Criteria for positive test**  Provocation of familiar neck, shoulder, or arm symptoms (neurological or pain) during the procedure, combined with structural differentiation through cervical side bending, release of shoulder girdle depression, or release of wrist extension, depending on the location of the provoked symptoms. |
